# Cosmopsychism and Consciousness Research: A Fresh View on the Causal Mechanisms Underlying Phenomenal States

**DOI:** 10.3389/fpsyg.2020.00371

**Published:** 2020-03-05

**Authors:** Joachim Keppler, Itay Shani

**Affiliations:** ^1^Department of Consciousness Research, DIWISS, Roth, Germany; ^2^Department of Philosophy, Sun Yat-sen University, Zhuhai Campus, Zhuhai, China

**Keywords:** neural correlates of consciousness, hard problem of consciousness, explanatory gap, ubiquitous field of consciousness, zero-point field, modulation mechanism, quantum systems, long-range coherence

## Abstract

Despite the progress made in studying the observable exteriors of conscious processes, which are reflected in the neural correlates of consciousness (NCC), there are still no satisfactory answers to two closely related core questions. These are the question of the origin of the subjective, phenomenal aspects of consciousness, and the question of the causal mechanisms underlying the generation of specific phenomenal states. In this article, we address these questions using a novel variant of cosmopsychism, a holistic form of panpsychism relying on the central idea that the universe is imbued with a ubiquitous field of consciousness (UFC). This field is understood as a foundational dual-aspect component of the cosmos, the extrinsic appearance of which is physical in nature and the intrinsic manifestation of which is phenomenological in nature. We argue that this approach brings a new perspective into play, according to which the organizational characteristics of the NCC are indicative of the brain’s interaction with and modulation of the UFC. Key insights from modern physics suggest that the modulation mechanism is identical with the fundamental mechanism underlying quantum systems, resulting in the conclusion that a coherently oscillating neural cell assembly acquires phenomenal properties by tapping into the universal pool of phenomenal nuances predetermined by the UFC, or more specifically, by entering into a temporary liaison with the UFC and extracting a subset of phenomenal tones from the phenomenal color palette inherent in the basic structure of the UFC. This hypothesis is supported by a substantial body of empirical evidence.

## Consciousness and the NCC

Neuroscientific approaches to the study of consciousness assign pride of place to the task of progressively charting and analyzing the *neural correlates of consciousness* (NCC), i.e., the neural mechanisms jointly sufficient for eliciting specific types of conscious experiences (Crick and Koch, 2003; Tononi and Koch, 2008). The search for the NCC is motivated, in large part, by the belief that they are *more* than mere correlates, namely, that such neural mechanisms provide the causal-material basis for consciousness. Significantly, many neuroscientists also hold (or have held), optimistically, that an increased knowledge of the NCC will eventually shed light on the fundamental riddle known as the *hard problem* of consciousness, namely, the problem of understanding what it is about the brain which enables it to generate something as remarkable and unique as subjective phenomenal experience. In short, from the point of view of conventional neuroscientific lore the activity patterns constituting the NCC are not just observable concomitants of subjective experience in highly complex animals, but, rather, the ultimate foundation of consciousness (see, e.g., Seth et al., 2006).

In identifying the NCC with the ultimate basis of consciousness, this conventional approach is beset by two cardinal deficiencies. First, it severely restricts the spectrum of the possible causal mechanisms underlying consciousness, a restriction questionable on both empirical and theoretical grounds. Second, it remains orthogonal to the concerns driving the hard problem, unable to address these concerns head-on.

Before we attend to these problems, it is worth recalling, first, that few would deny that consciousness, as manifested in humans and in other advanced animals such as primates, dolphins, or birds (for example), bears special connection to the brain, and in particular to the specific processes and activity patterns which neuroscientists identify as the NCC. However, notwithstanding that there is a special connection between consciousness and the brain, and notwithstanding the relevant neuroscientific evidence, the precise *nature* of this connection remains an open question. In particular, the idea that consciousness is identical to such brain processes, or that these processes generate consciousness from utter insentience, is an *interpretation* of the data—it is something which neither the phenomenal, nor the behavioral, nor the neurophysiological data necessitate. To be sure, knowledge of the NCC should inform and constrain our efforts to understand consciousness and to shed light on the nature of the psychophysical nexus, but it does not deliver ready-made answers to the above-mentioned core questions.

## Against an Unnecessary Limitation of Theoretical Horizons

Consider now the restriction of the spectrum of possible causal mechanisms underlying macro-scale phenomenal consciousness. One sense in which cerebral chauvinism is ill-advised is evinced in the accumulation of evidence suggesting that the bounds of consciousness in the living world may far exceed cranial circumscription. To begin with, some highly intelligent creatures such as octopuses and other cephalopods are endowed with large neural ganglia on their arms, supporting sophisticated forms of sensing and moving with significant degree of autonomy from the octopus’ brain (Hanlon and Messenger, 1996; Godfrey-Smith, 2013). More radically still, there is growing evidence for the existence of complex behavior in organisms lacking brains altogether. An intriguingly broad array of cognitive abilities is being progressively unveiled in simple eukaryotes, prokaryotes, and plants. Variegated forms of perception and behavioral plasticity, information processing, anticipation, memory, learning, valence, problem solving, communication, and cooperation are attributed to various brainless organisms from slime molds (Nakagaki et al., 2000; Reid et al., 2012), to bacteria (Ben-Jacob et al., 2006; Lyon, 2015), to plants (Trewavas, 2014; Gagliano, 2017).

In congruence with such studies, there is also a growing tendency to view neuronal networks as but one special case (albeit particularly powerful) of a general network dynamics whose fundamental principles are exemplified throughout the entire spectrum of biological life (Lyon, 2015; Baluŝka and Levin, 2016). In other words, many cognitive functions which in creatures such as Macaque monkeys, bees, or humans, are mediated through cerebral activity appear to be manifest, to some degree, in different forms of life (such as plants, slime molds, or bacteria) using alternative types of informational networks: be it methylation DNA networks, root systems, cytoskeletal elements, non-neural bioelectricity, calcium signaling, and so on.

While such studies often do not involve direct reference to consciousness (but see Trewavas, 2014; Baluŝka and Reber, 2019), the steady growth in evidence attesting to the existence of sophisticated cognitive repertoires throughout life’s spectrum puts increasing pressure on the orthodox notion that consciousness and the NCC are coextensive. As soon as we cease taking such coextension for granted, we enjoy greater freedom to consider a wider range of possible causal mechanisms as potential candidates for a comprehensive explanation of consciousness.

## Minding the Explanatory Gap

Another cause for skepticism regarding the view that the NCC provide the ultimate basis of phenomenal experience is the familiar hard problem of consciousness (Chalmers, 1995). For given any set of neural configurations proposed as a proper physical underpinning for consciousness, there remains the question *why* such configurations should culminate in subjective experience. In the words of some notable early observers, the chasm between the physical and the phenomenal (as these are canonically understood by science, philosophy, and commonsense) appears to be “intellectually impassable” (Tyndall, 1879, 18), with the result being that the hypothesis that experience comes about through the irritation of nervous tissue “is just as unaccountable as the appearance of Djin when Aladdin rubbed his lamp” (Huxley, 1866, 193).

Underlying this gap is a fundamental dichotomy between the objective and the subjective. Science approaches its objects of study from an objective, third-person, perspective. Its descriptions are confined to the outward appearance of things, even when such “things” (objects, processes, events, or mechanisms) unfold inside the body or brain. It concerns itself exclusively with the behavior and structure of causal agents, that is, with the observable exteriors of its target explananda. In contrast, consciousness is a subjective, first-person, phenomenon. Its inner presence constitutes a manifest immanent reality irreducible to observable behavior and structure. Thus, in any approach confined to externals consciousness is bound to remain alien: identified, perhaps, but neither fully assimilated nor properly explained. For this reason, it is imprudent to expect that more elaborate accounts of the neural basis of consciousness could ever be sufficient to address the challenge posed by the hard problem.

## Weaving Science and Philosophy Synergetically

What is needed, we submit, is a fresh outlook. Inclusive of consciousness and the intrinsic dimension of things but, at the same time, hospitable to objective findings and to rigorous scientifically based analysis. In this respect, the contribution of philosophy is vital. Philosophy’s quest is maximally comprehensive in that it seeks to understand reality as a whole. As such, it must take into consideration the outer as well as the inner dimension of things. Moreover, its ultimate goal of arriving at an integral picture of reality in its entirety implies a commitment to strive to make sense of the connection between these two complementary aspects—the objective and the subjective. Consequently, it has ample historical and conceptual resources to draw upon in the effort to contribute to an improved understanding of the psychophysical nexus. In particular, we believe that some philosophical ideas recently rediscovered and redeveloped within the fields of metaphysics and the philosophy of mind give fresh impetus to consciousness research in that they provide a conceptual matrix opening up new interpretations of the neuroscientific body of evidence and, potentially, leading to unprecedented research strategies. In this spirit, we present the central ideas behind a novel variant of *cosmopsychism*, a holistic form of panpsychism from the genus of priority cosmopsychism that relies on the assumption of a cosmic level of consciousness serving as the ultimate bedrock of experiential reality (Keppler, 2012; Shani, 2015; Shani and Keppler, 2018; for different variants of priority cosmopsychism, see Mathews, 2011; Goff, 2017; Nagasawa and Wager, 2017).

## The Cornerstones of a Novel Cosmopsychist Paradigm

**FIGURE 1:**
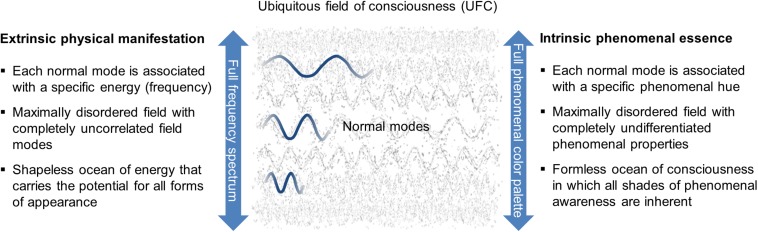
Dual-aspect character of the ubiquitous field of consciousness (UFC). It is postulated that the UFC is composed of a spectrum of normal modes that exhibit an extrinsic physical manifestation and an intrinsic phenomenal essence. From the physical perspective, each normal mode is associated with a specific energy and frequency. Due to basic symmetry requirements, the undisturbed ground state of UFC is assumed to be a maximally disordered field with completely uncorrelated field modes, or expressed differently, a shapeless ocean of energy that carries the potential for all forms of appearance. From the phenomenological perspective, each normal mode is associated with a specific phenomenal hue. Accordingly, the structure of the UFC ground state can be translated to the effect that we are dealing with a maximally disordered field with completely undifferentiated phenomenal properties, or put another way, a formless ocean of consciousness in which all shades of phenomenal awareness are inherent. Thus, what manifests extrinsically as the full frequency spectrum of the UFC represents intrinsically the full phenomenal color palette.

Our approach is based on the central idea that the universe is imbued with an *inherently sentient medium*, hereafter referred to as *ubiquitous field of consciousness* (UFC). In order to avoid substance dualism, which would immediately present us with the problem of causal interaction, we require this field to be seamlessly embedded in the edifice of modern physics. Consequently, we posit that the UFC is a foundational *dual-aspect* component of the cosmos, the extrinsic appearance of which is physical in nature and the intrinsic manifestation of which is phenomenological in nature (more on this in the concluding section). As with all fields that play a role in physics, the extrinsic nature of the UFC should reveal itself in energetic form, which is reflected in a spectrum of oscillations, the so-called normal modes. Moreover, it is to be expected that in its initial state the field satisfies all essential symmetry requirements (isotropy, homogeneity, scale invariance, Lorentz invariance), entailing that there is no preferential direction, location, and reference system. This leads directly to the conception of the undisturbed UFC as an omnipresent, formless ocean of activity with completely uncorrelated modes (Keppler, 2016, 2018a). We then postulate that each normal mode is associated with an elementary phenomenal hue, implying that the entire phenomenal “color palette” is represented by the oscillatory spectrum of the UFC, with the terms borrowed from color vision being understood as illustrative metaphors that we use here and in the following in a broader sense to cover the entire range of phenomenal qualities. Accordingly, from the phenomenological point of view, the ground state of the UFC can be described as a shapeless, undifferentiated ocean of consciousness in the basic structure of which all shades of phenomenal awareness are inherent (Keppler, 2012; Shani and Keppler, 2018). Even though, from the perspective of our paradigm, no concrete conscious state can be assigned to the maximally disordered ground state of the UFC, experiences collected in deep states of meditation suggest that this ground state may be characterized as a maximally unified phenomenal state (for a more detailed discussion, see Shani and Keppler, 2018). The ubiquitous background field thus constitutes an entity that embodies the principles of physics and at the same time contains within itself the phenomenological basis of ultimate reality (see Figure 1).

**FIGURE 2:**
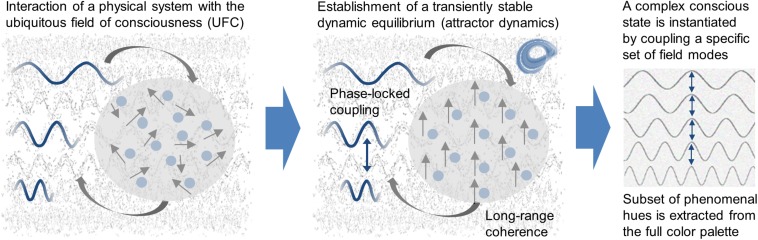
Causal mechanism underlying phenomenal states. It is postulated that conscious systems must be equipped with a fundamental mechanism by means of which they are able to influence the basic structure of the ubiquitous field of consciousness (UFC). This requires the interaction of a physical system with the UFC in such a way that a transiently stable dynamic equilibrium, a so-called attractor state characterized by long-range coherence, is established in which the involved field modes enter into a phase-locked coupling. Based on the conception that each field mode is linked to an elementary phenomenal hue, the coupling of a specific set of field modes results in the extraction of a subset of phenomenal hues from the full phenomenal color palette and, hence, in the instantiation of a complex conscious state.

Following this line of thought, it is natural to assume that conscious systems must be equipped with a fundamental mechanism by means of which they are able to influence the basic structure of the UFC, resulting in modified UFC states each of which displays complementary, intimately related physical and phenomenal properties. Without such a mechanism, there would be no conscious states other than the ground state of the UFC. Therefore, *a distinctive feature of conscious systems in comparison to non-conscious systems must be the capacity to modulate the omnipresent field of consciousness*, imposing constraints on the modulation mechanism. A look at the previously postulated properties of the UFC, according to which each normal mode of the field is linked to an elementary phenomenal hue, gives rise to the hypothesis that specific complex states of consciousness are formed by binding together specific sets of field modes. Consequently, using a rendering that is in accordance with modern physics, we argue that conscious states are caused by the dynamic interaction of a physical system with the UFC, provided that different modes, corresponding to the resonance frequencies of the system, are involved in this interaction and that the physical system establishes a transiently stable relationship with the background field resulting in the phase-locked coupling of the participating modes (Shani and Keppler, 2018). From this point of view, a physical system acquires phenomenal properties by entering into a temporary liaison with the cosmic field of consciousness and extracting a subset of phenomenal tones from the spectrum of all phenomenal tones potentially present in the field (see Figure 2). Conversely, this means that the phenomenal shades a given system can extract are determined by the range of dynamically stable states arising from the system-specific set of resonance frequencies. As a direct consequence, systems that cannot achieve dynamic equilibrium with the UFC have no access to its immanent phenomenal color palette and, hence, cannot generate phenomenal states (Shani and Keppler, 2018).

## The Novel Cosmopsychist Paradigm Meets Reality

It is now of vital significance that recent developments in modern physics are fully compatible with the above considerations. This applies particularly to stochastic electrodynamics (SED), “a branch of physics that affords a look behind the scenes of quantum mechanics and quantum field theory (QFT),” thereby “unveiling the mechanisms that account for the quantum behavior of matter” (Keppler, 2018a, 2). The foundations of SED, which date back to the 1960s, have been permanently refined in the endeavor to build a solid conceptual framework for quantum theory (Marshall, 1963, 1965; Boyer, 1969, 1975; De la Peña and Cetto, 1994, 1995, 1996, 2001, 2006; De la Peña et al., 2009, 2015). A pivotal ingredient of this framework is “an all-pervasive electromagnetic background field, called zero-point field (ZPF), which, in its original form, is a homogeneous, isotropic, scale-invariant, and maximally disordered ocean of energy with completely uncorrelated field modes and a unique power spectral density” (Keppler, 2018a, 2). Based on this idea, the key findings of SED may be summarized to the effect that

1. “Every material system can be regarded as an open stochastic system in permanent contact with the random ZPF” (Keppler, 2018a, 2),
2. The dynamic interaction between a physical system and the ZPF can achieve energetic equilibrium, given that “the interaction strength between the oscillating components and the relevant field modes, for which the system exhibits a strong resonant behavior, exceeds disturbing forces, such as thermal noise” (Shani and Keppler, 2018, 397),
3. “A system in equilibrium with the ZPF falls into a dynamically stable state, that is, an attractor, and displays *quantum behavior*” (Shani and Keppler, 2018, 397),
4. “The orchestration of an attractor requires the initially chaotic ZPF to change over to a partially ordered state that is characterized by an attractor-specific set of phase-locked field modes” (Keppler, 2018a, 2), which has the consequence that “all the components of the system are effectively coupled through the ZPF, giving rise to collective cooperation and *long-range coherence*” (Shani and Keppler, 2018, 398).

In light of these insights, “SED commends itself as a promising approach for the integration of consciousness into a coherent theoretical framework” (Keppler, 2016, 352). In particular, the findings listed above “suggest that the ZPF is perfectly suited for playing the dual role as the carrier of both primordial energy and consciousness” (Shani and Keppler, 2018, 399), which amounts to identifying the UFC with the ZPF. Moreover, in view of the previously formulated postulates relating to phenomenal states, the discoveries of SED support the assertion that “the mechanism underlying quantum systems has all the makings of a truly fundamental mechanism behind conscious systems, leading to the assumption that *conscious systems extract their phenomenal qualities from the phenomenal color palette immanent in the ZPF*” (Keppler, 2018b, 3). As a derivation, “conscious systems can be expected to display quantum behavior,” meaning that “*the formation of transiently stable coherent states is an essential prerequisite for conscious awareness*” (Keppler, 2018b, 3).

Exactly this expectation is confirmed, especially as “the currently available body of evidence and the entirety of observations suggest that the brain has all the characteristics of a macroscopic quantum system” (Keppler, 2013, 3), which is substantiated in the following. Limiting ourselves for the moment to conscious perception, it is widely accepted “that the NCC are related to large-scale synchronization in the brain” (Keppler, 2018b, 3), a conclusion that is based on a considerable amount of neurophysiological data (Crick and Koch, 1990; Desmedt and Tomberg, 1994; Rodriguez et al., 1999; Engel and Singer, 2001; Melloni et al., 2007; Doesburg et al., 2009; Gaillard et al., 2009; Singer, 2015). A closer examination of the data (Freeman, 1991, 2004, 2005, 2007, 2009) reveals “that the NCC can be equated with attractors distinguishing themselves by a high degree of coherence between spatially distributed cortical areas and that our streams of conscious perception are based on the recurring formation and dissolution of such coherent states” (Keppler, 2018b, 3). These insights corroborate the assertion “that the NCC bear on quantum coherence since a rigorous description of the observed features, such as macroscopic pattern formation and second-order phase transitions, requires the formalism of quantum physics” (Keppler, 2018b, 3), which was also clearly emphasized by Freeman and Vitiello (2006, 2007). Including the previously enumerated findings of SED, this indicates “that the ZPF is involved in the orchestration of coherent neural activity patterns” (Keppler, 2018b, 3) and that “*the brain produces an individual stream of consciousness by periodically modifying the ZPF*” (Keppler, 2013, 3). It should be pointed out that this self-consistent explanatory approach can be extended beyond conscious perception to incorporate also self-referential consciousness (Keppler, 2018b), altered states of consciousness (Keppler, 2018a, b), as well as declarative memory functions (Keppler, 2020).

## Conclusion and Outlook

The strength of the novel cosmopsychist paradigm presented here lies in the bridging of the explanatory gap the conventional materialist doctrine struggles with. This is achieved by proposing a comprehensible causal mechanism for the formation of phenomenal states that is deeply rooted in the foundations of the universe. More specifically, the sort of cosmopsychism we advocate brings a new perspective into play, according to which the structural, functional, and organizational characteristics of the NCC are indicative of the brain’s interaction with and modulation of a UFC. In this respect, the key insights from SED suggest that this field can be equated with the ZPF and that the modulation mechanism is identical with the fundamental mechanism underlying quantum systems, resulting in our conclusion that a coherently oscillating neural cell assembly acquires its phenomenal properties by tapping into the universal pool of phenomenal nuances predetermined by the ZPF. This hypothesis is supported by a large body of empirical evidence.

The novel cosmopsychist paradigm elegantly circumvents the hard problem that arises in prevailing materialist approaches because there are “principled reasons to doubt that phenomenal facts are necessitated by purely structural (or functional or organizational) facts” (Shani and Keppler, 2018, 406). The crucial difference is that in our approach “the relevant structural facts … are tasked not with the generation of experience *per se* but, rather, with its modulation and restricted expression” (Shani and Keppler, 2018, 406), leading to well-defined distinctive features between conscious and non-conscious systems as well as conscious and unconscious brain processes. In this context, it should be highlighted that the proposed causal mechanism underlying phenomenal states is predicted to be accompanied by a concomitant phenomenon, namely the emission of characteristic photon pulses (Keppler, 2016, 2018b), paving the way for a new research strategy that aims at corroborating the hypotheses formulated in this paper and eventually ends in the systematic “derivation of psychophysical mapping rules between particular qualia and particular sets of phase-locked ZPF modes” (Shani and Keppler, 2018, 407).

Finally, a note is appropriate with regard to the causal interpretation of our UFC account. According to our approach, the UFC has two complementary description levels (hence the earlier reference to a double-aspect perspective), each of which is coherent in itself. From the physical perspective, the dynamic interaction of the UFC with material systems can be consistently described in terms of energy transfer ensuring causal closure and energy conservation, so that the evolution of the UFC is fully determined by its physical properties. On this reading, the evolving field passes through a series of physical states and the phenomenal qualities associated with each state can be regarded as accompanying features of the physical processes. On the other hand, even though there remains certainly a lot of work to be done, we believe that our approach has the potential to set the stage for a phenomenological interpretation of dynamical processes, meaning that the processes of which we usually think in terms of physical causation may be self-consistently interpreted in conceptually alternative, phenomenal terms. From this point of view, consciousness may be causally efficacious and turn out to be the ultimate intrinsic force underlying the dynamic transformations described by physics, thus laying the foundations for a scientifically informed idealist worldview.

## Author Contributions

Both authors contributed equally to this work and approved it for publication.

## Conflict of Interest

The authors declare that the research was conducted in the absence of any commercial or financial relationships that could be construed as a potential conflict of interest.
